# The Role of Patient-Specific Morphological Features of the Left Atrial Appendage on the Thromboembolic Risk Under Atrial Fibrillation

**DOI:** 10.3389/fcvm.2022.894187

**Published:** 2022-07-14

**Authors:** Giulio Musotto, Alessandra Monteleone, Danila Vella, Sofia Di Leonardo, Alessia Viola, Giuseppe Pitarresi, Bernardo Zuccarello, Antonio Pantano, Andrew Cook, Giorgia M. Bosi, Gaetano Burriesci

**Affiliations:** ^1^Bioengineering Unit, Ri.MED Foundation, Palermo, Italy; ^2^Department of Engineering, University of Palermo, Palermo, Italy; ^3^UCL Institute of Cardiovascular Science and Great Ormond Street Hospital for Children, London, United Kingdom; ^4^UCL Mechanical Engineering, University College London, London, United Kingdom

**Keywords:** left atrial appendage, fluid–structure interaction, LAA morphology, patient-specific models, atrial fibrillation (AF)

## Abstract

**Background:**

A large majority of thrombi causing ischemic complications under atrial fibrillation (AF) originate in the left atrial appendage (LAA), an anatomical structure departing from the left atrium, characterized by a large morphological variability between individuals. This work analyses the hemodynamics simulated for different patient-specific models of LAA by means of computational fluid–structure interaction studies, modeling the effect of the changes in contractility and shape resulting from AF.

**Methods:**

Three operating conditions were analyzed: sinus rhythm, acute atrial fibrillation, and chronic atrial fibrillation. These were simulated on four patient-specific LAA morphologies, each associated with one of the main morphological variants identified from the common classification: chicken wing, cactus, windsock, and cauliflower. Active contractility of the wall muscle was calibrated on the basis of clinical evaluations of the filling and emptying volumes, and boundary conditions were imposed on the fluid to replicate physiological and pathological atrial pressures, typical of the various operating conditions.

**Results:**

The LAA volume and shear strain rates were analyzed over time and space for the different models. Globally, under AF conditions, all models were well aligned in terms of shear strain rate values and predicted levels of risk. Regions of low shear rate, typically associated with a higher risk of a clot, appeared to be promoted by sudden bends and focused at the trabecule and the lobes. These become substantially more pronounced and extended with AF, especially under acute conditions.

**Conclusion:**

This work clarifies the role of active and passive contraction on the healthy hemodynamics in the LAA, analyzing the hemodynamic effect of AF that promotes clot formation. The study indicates that local LAA topological features are more directly associated with a thromboembolic risk than the global shape of the appendage, suggesting that more effective classification criteria should be identified.

## Introduction

The risk of clot formation, ischemic events, and their neurological consequences (1, 2) increase significantly with the pathological condition of atrial fibrillation (AF), a cardiac arrhythmia characterized by irregular electrical activity due to the presence of multiple trigger points on the atrial surface. Although the pathogenesis is still unknown, a vast majority of intracardiac thrombi detected in patients with AF were observed in the left atrial appendage (LAA) (3). This is an anatomical structure departing from the left atrium (LA), partly leaning on the outer wall of the left ventricle, that in normal conditions contracts actively (4) in phase with the atrium. It is reported to play a role in the pressure regulation of the LA, acting as a decompression chamber during the pressure peak corresponding to the ventricular systole (5) and releasing atrial natriuretic peptide (ANP), which can normalize atrial pressure through the activation of specific receptors (6). The LAA is currently classified into four main morphological variants, commonly identified on the basis of the resembling shape as follows: *chicken wing*, *windsock*, *cactus*, and *cauliflower*. Possible relation between the morphological classification and the thrombo–embolic risk has been widely investigated in the literature (7, 8), and a number of numerical studies have been performed on patient-specific models, aiming at identifying hemodynamic changes produced by AF that can be related to higher clotting (9, 10). In particular, the blood shear strain rate (SSR) is a fluid dynamic parameter that can be easily estimated from computational analyses and is commonly correlated with thromboembolic risk (9, 11–14). In fact, SSR is directly related to blood stagnation, which impairs the balance between procoagulant biochemical species, such as thrombin, and anticoagulants promoting the accumulation of the former and the reduction of the latter (15). Moreover, low shear conditions favor platelet activation, adhesion, and stabilization of platelet aggregates, enhancing platelet–fibrin and platelet–endothelium interactions (16).

Still, the numerical studies available in the literature often model the appendage walls as rigid, thus neglecting the contractility and the alteration of the physiological properties and geometric features produced by persistent AF conditions (10, 17). Other studies have included pathological alterations in terms of contractility and remodeling, clearly indicating that these factors can play a major role in the fluid dynamics in the LAA and its alterations produced by AF (14). However, these were applied to simplified anatomical models. This work analyses four patient-specific geometrical models of LAA, classified according to the four morphological variants (9) by means of computational fluid–structure interaction (FSI) simulations.

The models include the active and passive contractility of the LAA walls in conditions of sinus rhythm and AF conditions and simulate their remodeling expected after persistent AF (18, 19). The aim of the work is to clarify the mechanisms related to AF that promote clot formation, and their potential association with anatomical phenotypes and hemodynamic parameters, which could suggest more effective stratification approaches to the thromboembolic risk.

## Materials and Methods

### Models Design

The left atrium is characterized by very complex flow patterns, which depend on the number, shape, and position of the pulmonary veins; the location and shape of the mitral valve; the LAA orifice; and the pressure and flow conditions acting at the veins and valvular ostium (20, 21). Hence, to isolate the role of the atrial appendage and avoid introducing the effect of the patient-specific features characterizing the surrounding anatomy, only the appendage was modeled and analyzed in this work. This assumption does not produce substantial approximations in the determined flow, especially in the distal regions, where the effect of the atrial flow regime becomes negligible and the presence of fluid stagnation increases the risk of clot formation (9).

During the cardiac cycle, the LAA walls deform under the cyclic mechanical actions exerted by blood, while influencing the hemodynamics as a consequence of its passive movement and active contraction. Hence, a two-way FSI simulation was preferred, where the fluid and structural domains are solved in parallel and converge at each step.

A commercial software Ansys 19.2 was used for the simulations. The structural domain was analyzed using an implicit time integration scheme in the Ansys Transient Structural module, as recommended for static and low-frequency dynamic analyses (22, 23). Despite involving a computationally expensive inversion of the global stiffness matrix, these integration schemes have the advantage of achieving global equilibrium at each iteration. Hence, their solutions are unconditionally stable and accurate also for relatively coarse time steps. The fluid domain is solved by employing the computational fluid dynamic (CFD) package Ansys CFX. This code adopts a vertex-based finite volume method to solve the Navier-Stokes equations in an Eulerian description. This approach is particularly convenient when dealing with unstructured tetrahedral meshes, as it strongly reduces the number of degrees of freedom and computational footprint compared to cell-based solvers (24). Moreover, the position of the control volume centered at the boundaries facilitates the treatment of boundary conditions (25) and their exchange at the interface between the two domains. The structural and fluid dynamics solutions were coupled in a two-way FSI simulation through the System Coupling available in the Ansys workbench, which facilitates the data transfer between individual single-physics solvers (26).

**FIGURE 1:**
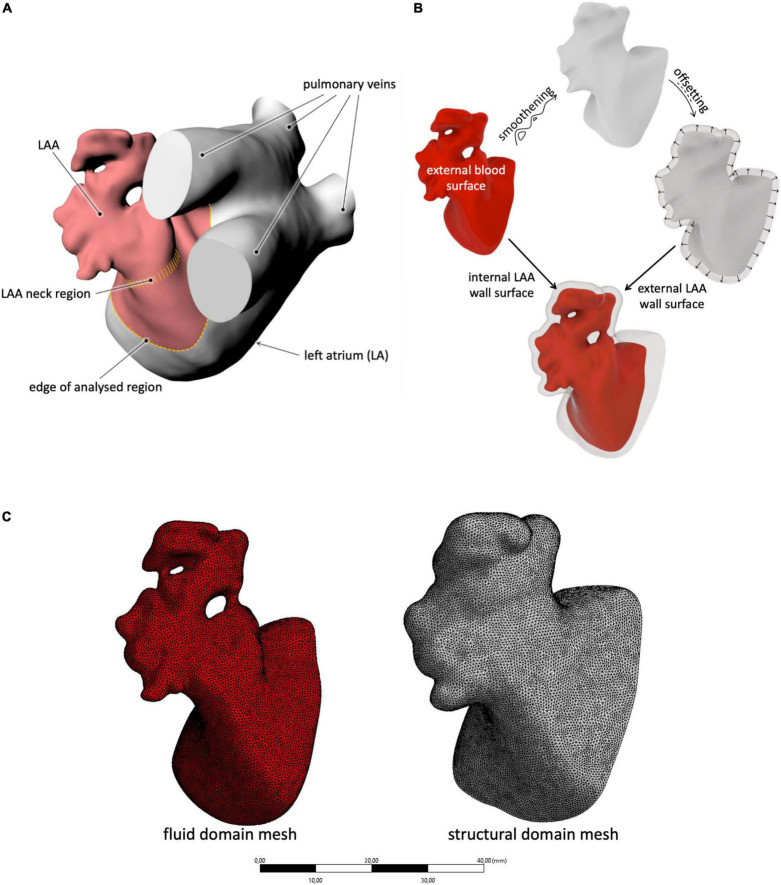
**(A)** Description of the selection method adopted for the LAA study region; **(B)** schematic procedure used to generate the inner and outer LAA walls, starting from the external blood surface; and **(C)** example of mesh for both the solid and fluid domains with the selected size.

The analyzed LAA geometries were defined starting from patient-specific morphologies used in the work by Bosi et al. (9). These are based on radiological scans (computed tomography scan) that return the blood content internal to the LAA and represent the four typical morphologies of the adult population not affected by AF. A detailed description of the segmentation process is provided in Bosi et al. (9). The considered portion of the LAA included the neck and a portion of the atrium between the proximal part of the LAA and the pulmonary veins (Figure 1A). This moves the boundary conditions away from the LAA orifice, allowing the contraction of the whole LAA (including its neck region) and the establishment of higher freedom to the fluid parameters at the LAA inlet. The obtained morphologies were processed on the open-source *Autodesk* program *MeshMixer*, where they were re-meshed and their imperfections were corrected.

The obtained models were closed with a bulge at the open inlet, obtained from a non-uniform rational basis spline (NURBS) tangent to the wall surface at the orifice. This acted as an open surface for the application of the fluid boundary conditions.

To generate the external surface of the LAA solid structure, the internal wall surface (corresponding to the external blood surface) was first smoothened to remove all discontinuities due to the presence of the trabecule and offset in the external direction by 2.1 mm (see Figure 1B). This thickness was selected on the basis of the average wall thickness measured in ovine models (27) and used as an external surface in the construction of the solid part. In particular, the internal and external meshes were transformed into poly surfaces and integrated into a solid volume through the commercial computer-aided design (CAD) software *Rhinoceros 7.0*.

As mentioned above, chronic AF is typically associated with enlargements in the LAA volume. Although the increase of volume associated with this condition is well documented and quantified, no information on the anatomical changes associated with this remodeling is found in the literature. Hence, to verify potential fluid dynamic changes produced by persistent AF, enlarged versions of the four LAA models were created by scaling them up by 150% (28). Despite this being a basic assumption, it is useful to analyze the effect of the LAA size, without introducing other spurious effects.

**FIGURE 2:**
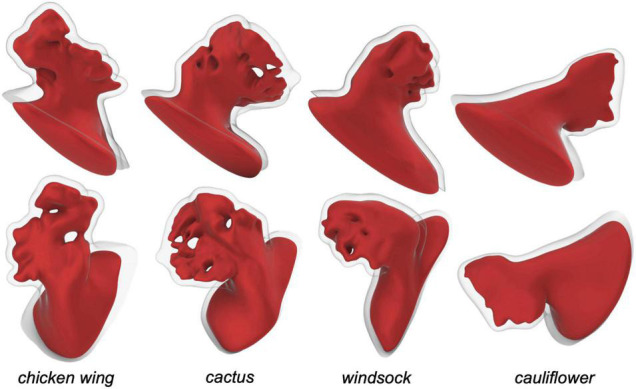
Left atrial appendage CAD models obtained for the four different patient specific shapes, seen from two views.

The CAD models were imported in *ANSYS Workbench* and meshed with quadratic tetrahedral elements for the fluid part and with linear tetrahedral elements for the structural part. The mesh density was selected on the basis of a convergence analysis performed on the *chicken wing* morphology, selected for the setup of all parameters as reported to be the most common, observed in nearly 50% of the patient’s population (29). In particular, an average mesh density of 70 element/mm^3^ was used for both the structure and the fluid (see Figure 1C). The four models are represented in Figure 2.

### Rheological and Mechanical Properties

Human blood is a concentrated suspension of deformable and aggregable cellular elements in plasma. This can lead to a non-Newtonian shear thinning behavior characterized by thixotropic and viscoelastic properties (30–32). Despite the availability of a number of non-Newtonian models, these are based on steady-state studies (33). Therefore, they are inadequate to describe blood rheology in pulsatile conditions due to the strong time-dependency of the cell aggregation mechanism. Hence, in this work, it was preferred to model the fluid as Newtonian, with a density of 1,062 kg/m^3^ and a viscosity of 0.0037 Pa⋅s, replicating the common physical properties of human blood at large shear rates. The Newtonian behavior is a common assumption that enables direct comparison of the presented work with similar studies in the literature while providing conservative results. In fact, regions, where prolonged stasis is detected, are likely to be even more stagnant under *in vivo* conditions, due to red blood cell aggregation and thrombus formation (34).

The tissue of the LAA wall exhibits reduced anisotropy, and the typical non-linearly stiffening constitutive behavior observed in biological soft tissues (27), is characterized by a low-modulus region, a transitional region characterized by a progressive stiffening with strain increases, and a stiff region at larger strains. However, the clinical morphological scans were acquired from pressurized operating hearts, already partially stressed/strained, hence, the wall material was modeled as linear elastic. Anisotropy is reported to be reduced in the wall of the atrium and its appendage (27). Hence, for simplicity, the material was modeled as isotropic. The Young’s modulus was set equal to 1.5 MPa, this being the intermediate between that reported for the low and high regions in the left atrium of porcine hearts (35). To account for the material incompressibility, a Poisson’s ratio of 0.49 was selected. During LAA contraction, the volume of the appendage is reported to reduce by approximately 60% of its maximum value as a combined effect of the tissue elastic response to the pressure variation and the active contraction of the wall muscle (36). Therefore, a uniformly distributed pressure was imposed at the open surface of the fluid of the *chicken wing* model, replicating the physiological atrial pressure curve obtained from the Wiggers diagram, and the change of volume due to the muscular contractive action was obtained through the setting of a virtual contraction of the structural part. This was achieved by defining orthotropic thermal expansion coefficients of the structural elements. In particular, a local reference system was imposed for each element of the mesh, with the *x-y* plane lying parallel to the inner wall. The coefficient of thermal expansion along the wall thickness (local direction *z*) was defined to maintain volume conservation (for any contraction along the plane there is a corresponding expansion along the thickness), thus mimicking the cardiac muscular behavior. Then, the virtual change of temperature applied to the structure to simulate active contraction was adjusted to replicate clinical evaluations of filling and emptying volumes of the LAA in healthy conditions (36) for the *chicken wing* morphology. In particular, the coefficients of thermal expansion were set equal to 0.05 in the in-plane directions (*x* and *y*) and equal to −0.10 in the out-plane-direction (*z*); and the temperature curve was varied between 0 and 5°C, so as to achieve a relative volume change during the cycle of approximately 60%. For the FSI simulations, the timestep used is 21.5 ms.

**FIGURE 3:**
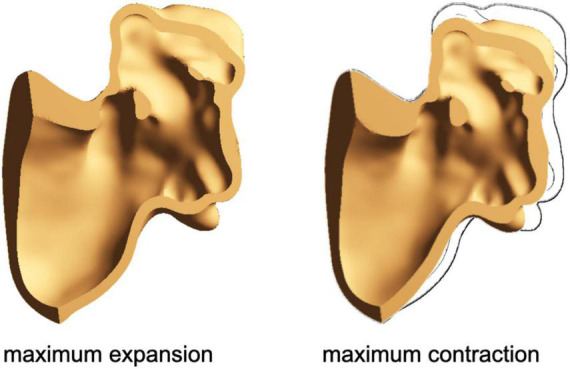
Cross-section of the chicken wing model at the maximum expansion **(left)** and at the maximum contraction **(right)**.

In Figure 3, a cross-section of the *chicken wing* structural domain model at the maximum expansion and the maximum contraction is represented. The same material parameters were applied to all models.

### Boundary Conditions

All nodes of the LAA proximal cross-section were fully constrained. Although this limits the ability of this region to follow a physiological dynamic, the fixed region is in the atrium and far from the neck of the LAA, which remains totally free to contract.

**FIGURE 4:**
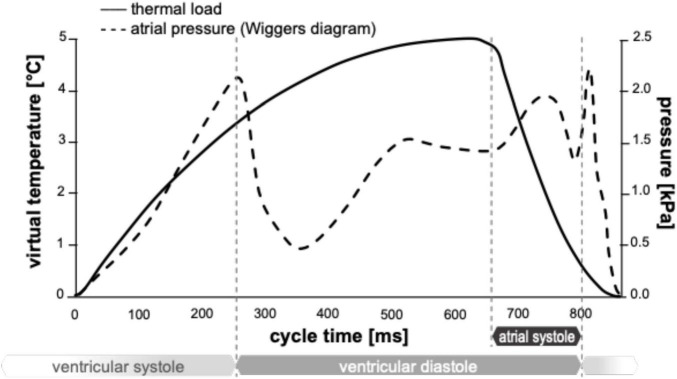
Thermal load (continuous line) and physiological atrial pressure curve (dashed line).

For the simulation of sinus rhythm conditions, a uniformly distributed pressure was applied at the open surface of the fluid, based on the physiological atrial pressure described in the Wiggers diagram (37, 38). Then, as described above, a virtual change of temperature was applied to the structure to simulate active contraction (see Figure 4).

For the simulation of acute and chronic AF conditions, no active contraction was enforced (by applying a constant virtual temperature equal to 0°C), and the pressure curve applied at the fluid-free surface was modified to account for the lack of atrial contraction (39–42). Hence, the structure only undergoes a passive expansion due to the pressure curve imposed at the opening surface of the fluid. In the case of chronic AF, typically associated with remodeling, the enlarged version of the appendage was used. The simulated heart rate was 70 beats/min, corresponding to a duration of each cycle equal to 860 ms. This is an idealized condition in the case of AF, which was characterized by irregular rhythm and was adopted to allow direct comparison of the results, isolating the effect of the loss of contraction and remodeling. Up to six cardiac cycles were simulated for each morphology, and stabilization of flows was detected for all cases as early as the second cycle.

## Results

In total, four different patient-specific models associated with the different morphological classes of the LAA (*chicken wing, cactus*, *windsock*, and *cauliflower*) were analyzed. For each patient-specific morphology, three conditions were analyzed: sinus rhythm, acute AF, and chronic AF, for a total of 12 different numerical simulations.

The volume changes obtained for the different cases are summarized in Table 1. The average of the volume variations for the four morphologies in sinus rhythm agrees with the physiological reference value used in the model calibration, as defined by Li et al. (36).

**TABLE 1:** Percentage volume change simulated for all models and all operating conditions.

	Sinus rhythm	AF acute	AF chronic
Chicken wing	56.15%	3.24%	3.30%
Cactus	57.86%	4.10%	4.46%
Windsock	57.43%	2.50%	2.77%
Cauliflower	74.12%	3.84%	3.85%

**FIGURE 5:**
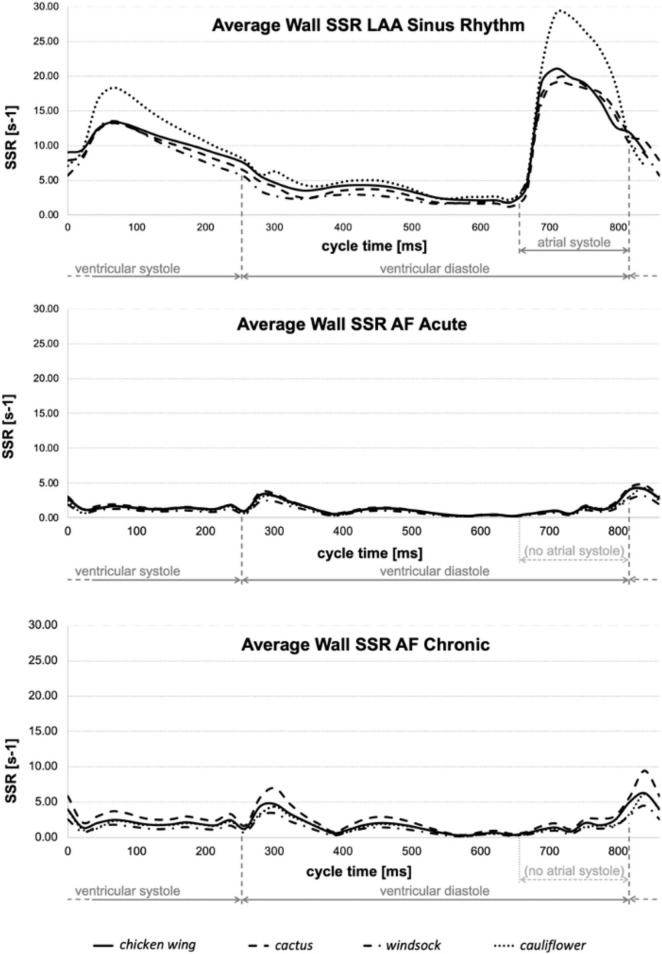
Average wall SSR estimated for all models in sinus rhythm **(top)**, acute AF **(middle)**, and chronic AF **(bottom)** conditions.

The physical quantities analyzed in the models are the SSR and the peak of velocity at the LAA orifice. As all analyses become periodical after the second cycle, the results were evaluated over the third cardiac cycle. The diagrams in Figure 5 represent the average wall SSR calculated in the region from the neck of the LAA (smallest orifice cross-section) to its distal end during the cardiac cycle for the four appendages in the three different conditions. For clarity, the time in the diagrams is relative to the represented cycle (goes from 0 to 860 ms) rather than to the analysis time (from 1,720 to 2,580 ms).

**FIGURE 6:**
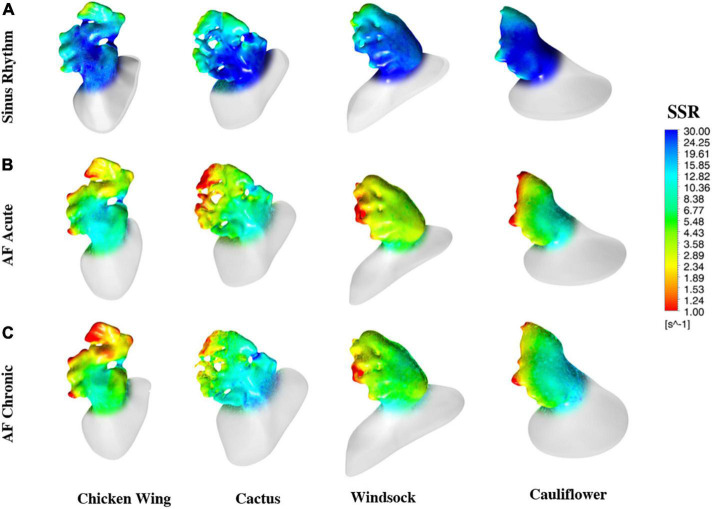
Color maps of the maximum wall SSR at the instant of the cycle when they reach the maximum value, estimated for all models and all operating conditions.

The color maps in Figures 6A–C represent the distribution of wall SSR at the respective peak of the average wall SSR identified from the diagrams in Figure 5. To support a more effective visualization of the range of wall SSR, a logarithmic rainbow scale is used in the pictures, with the red color indicating the regions of lowest shear rate where blood clotting is promoted.

**FIGURE 7:**
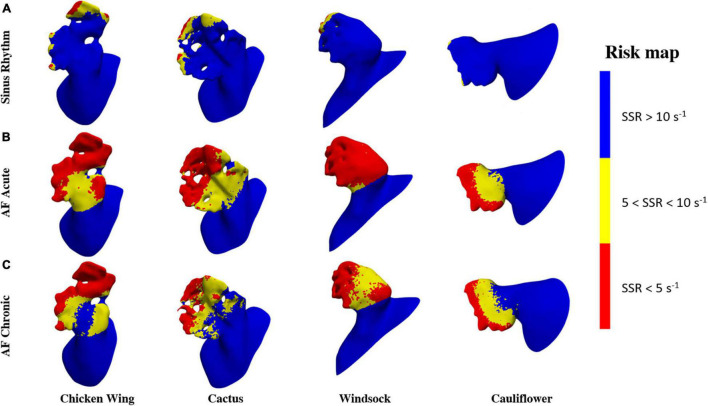
Risk area maps in **(A)** sinus rhythm, **(B)** AF acute, and **(C)** AF chronic.

To identify regions of the potential risk of thrombosis, the fluid wall that remains exposed for the entire duration of the cycle to SSR values below 10 and 5 s^–1^ are represented in Figure 7. These thresholds were selected to better identify the regions of low and very low persistent shear rates, where red blood cell aggregation is expected to be more pronounced with a consequent increase in viscosity (in the real case) and clotting (43).

For the different simulations, the percentage of LAA area exposed to SSR values below 10 and 5 s^–1^ for the entire cycle is summarized in Table 2.

**TABLE 2:** Percentage of LAA area exposed to SSR values below 10 and 5 s**^–^**^1^ for the entire cycle for the different operating conditions and all models.

	Sinus rhythm		AF acute		AF chronic	
	<5 s^–1^	<10 s^–1^	<5 s^–1^	<10 s^–1^	<5 s^–1^	<10 s^–1^
Chicken Wing	3%	17%	64%	93%	52%	76%
Cactus	3%	11%	55%	93%	33%	63%
Windsock	4%	17%	86%	100%	60%	96%
Cauliflower	0	3%	64%	95%	46%	79%

In the clinical setting, velocity remains the most used parameter for the purposes of a hemodynamic assessment (44). Hence, the maximum velocity predicted during the cycle at the LAA orifice cross-section for the different simulations was determined and reported in Table 3.

**TABLE 3:** Maximum velocity in sinus rhythm, AF acute, and AF chronic [cm/s].

	Sinus rhythm	AF acute	AF chronic
Chicken Wing	15	2.5	4.6
Cactus	10	2.8	4.4
Windsock	15	2.2	3.5
Cauliflower	20	1.9	4.7

## Discussion

Although the contraction parameters were set for the *chicken wing* model, under sinus rhythm conditions they produce a very similar percentage of volume changes in all models (about 57%), with the exception of the *cauliflower*, which experienced volume changes of around 30% higher (see Table 1). The different behavior of this model could be associated with the fact that it was the only one where the trabecular structure was absent. These may act as tie beams connecting the two main walls defining the appendage chamber, thus reducing the ability of the LAA to modify substantially its shape under the effect of the internal and external loads. These differences are well reflected in the average wall SSR in the LAA region, which is very similar to the *chicken wing, cactus*, and *windsock* models during the whole cardiac cycle, peaking during the active contraction to values around 20 s^–1^ and looks amplified by about 30% for the *cauliflower* (see Figure 5, Top).

Under acute and chronic AF conditions, the volume changes reduce dramatically for all cases by over an order of magnitude, with the *cactus* model exhibiting the largest volume change and the *windsock* the lowest, for both acute and chronic conditions (see Table 1). Volume variations for the chronic models were expectedly slightly larger, due to the stronger displacements produced by the same pressure acting on larger sections. Interestingly, although the average wall SSR in the LAA region still appears to relate well with the volume change for each condition, it only reduces to about one-fifth of the one predicted in sinus rhythm in the acute case, and to one-third in the chronic case. Globally, the levels of SSR predicted from the models compare well with those indicated in Vella et al. (14), although that study identified higher values observed in the acute conditions than in the enlarged chronic condition. This discrepancy may be because, in Vella et al. (14), LAA wall displacements were imposed, limiting the interplay between the different wall extensions and the elastic expansion and recoil of the structure. This suggests that FSI approaches are more adequate to capture the different factors controlling the phenomenon.

A comparison of the diagrams in Figure 5 for sinus rhythm and AF conditions indicates that the increase in wall SSR is not just associated with the active contraction producing the atrial systole, but the consequent relaxation phase is equally important, producing an increase in the SSR lasting for the ventricular systole. During this phase, when there is no flow leaving the atrial chamber through the mitral valve, the relaxation of the LAA (a similar effect can be expected for the whole atrial chamber) produces a flow that prevents blood stasis and all potential associated risks.

The color maps of wall SSR distribution (see Figure 6) indicate that, in normal conditions, the shear rate reduces at the distal edges and, in particular, after the trabecule and at the lobes. Knees, such as the abrupt bends characterizing the main body of the *chicken wing* and *windsock*, are also associated with some reduction in shear rate. These effects become more pronounced and extended in AF conditions, especially under acute conditions, maintaining the same spatial distribution.

The risk maps in Figure 7 offer a clearer visualization of the regions where flow conditions promoting blood thickening and clotting are more likely to occur. These risk regions are extremely reduced (less than 5% of the LAA surface is constantly below 5 s^–1^) and confined at the distal portion of the lobes for the healthy case, but occupy over 55% under acute AF conditions.

This confirms that the active contraction of the LAA plays a clear and marked role in the correct hemodynamics of the region, allowing adequate washing and reducing the risk of clot formation. The maps of risk also show very clearly the crucial role of the lobes (Figure 7A) and knees (see yellow regions in Figure 7B) in establishing low shear rate regions.

The general behavior appears to be very homogeneous for all models, with the exception of larger volume changes during the cycle and wall SSR predicted for the *cauliflower* in sinus rhythm conditions (this morphology is infrequent, and reported in only about 3% of patients according to Di Biase et al. (29). Under AF conditions, despite the significant morphological differences, all models are well aligned in terms of shear rate values and predicted level of risk.

The association of the LAA morphology with the insurgence of ischemic events is still controversial, with the chicken wing anatomy alternatively identified as the safest one (29, 45) or as the one associated with higher risks (46). Other reports indicate the cauliflower type as a potential predictive risk factor (47, 48). The presented study justifies the incoherency of these findings, diminishing the role of the current morphological classification and attributing more relevance to local topological features, such as the number and depth of lobes, the presence of bends, and the extent of trabeculation. This is supported by a number of clinical studies (48–50) and indicates the need for a new classification, better related to anatomical characteristics that influence more directly the hemodynamics within the LAA.

Velocities predicted at the orifice were lower than those clinically detected in clinical studies (44). However, direct comparison is not possible, as the velocity measured in patients from echocardiography also includes the motion of the orifice as the effect of atrial motion, which is not taken into consideration in the presented model. It is interesting to observe that, although velocities drop substantially as an effect of AF (Table 3), no direct relation between the velocity magnitude and the estimate of the risk can be found. In fact, the orifice velocity depends on the rate of volume variation and the orifice cross-section, so that it is not directly related to the hemodynamics that establishes in the distal portion of the appendage.

The study is based on a number of assumptions and simplifications on the modeled region, the boundary conditions, the material properties, and the rheological characteristics of blood. Although analyzing only the atrial appendage may neglect the effect of the complex fluid dynamics established in the left atrium, the effect of this contribution over the velocities and shear rates estimated on the LAA from previous studies typically dissipates in the first third of the appendage (9, 14). Moreover, the flow patterns in the atrium strongly depend on a number of boundary conditions, such as the pressure at each pulmonary vein or the flow through the mitral valve, whose modeling requires further assumptions and uncertainties. The wall tissue was modeled as linear elastic, with Young’s modulus intermediate between that in the low and high rigidity regions in animal tests. Although this choice simplifies the study and avoids substantial alterations of the patient-specific morphologies, the compliance of the atrial wall directly contributes to the component of volume change associated with a passive response to pressure. Hence, its role on the behavior under AF conditions can be relevant. Moreover, it is not known if and how this parameter changes during remodeling. The wall thickness was selected on the basis of animal data and kept constant for all models. The Young’s modulus and wall thickness attributed to the LAA structure necessarily have an influence on the passive emptying and filling of the LAA. Lower Young’s moduli and thinner walls would increase the fluid pressure contribution compared to the active contraction component. In AF conditions, where the active contraction is missing, modifying these parameters would result in changes in volume variations and SSR values, potentially affecting the evaluation of the thromboembolic risk. Similarly, the passive phases are also directly influenced by the pressure curve imposed at the inlet of the fluid domain, with filling and emptying volumes increasing with the mean and amplitude of the applied pressure load. For what concerns the active phase, the electro-mechanical coupling which characterizes the muscle action was emulated by applying a thermal contraction, following the procedure proposed by Carmody et al. (51). In this case, the thermal response of the material was set to accompany the contraction in a specific direction with dilatation in the orthogonal directions, similar to muscle behavior. Changes in these parameters would alter the effect of the active emptying and the corresponding filling phases. Finally, blood was assumed as Newtonian, thus underestimating the increase of viscosity in the regions of prolonged low shear rate. As mentioned above, this is a conservative assumption, which underestimates the predicted stagnation and clotting potential.

All parameters described above were set on the basis of the available sparse information found in literature and were applied unchanged to all models. This has allowed for the replication, a physiological behavior consistent with clinical observations, providing a better understanding of the phenomena studied. However, the availability of more specific data in healthy and patients with AF about the LAA pressures and dynamics, and the characterization of post-mortem human samples will be essential to increase the reliability of future models.

## Conclusion

This work analyses four patient-specific LAA anatomies belonging to different morphological types to investigate the mechanisms that promote clot formation in AF conditions. The analysis was performed by means of FSI simulations, modeling the active and passive contractility of the LAA walls in sinus rhythm, and in the/AF condition, also replicating the LAA remodeling typically caused by persistent AF.

The study confirms that the active contractility of the LAA muscular wall is essential in ensuring a physiologically healthy flow, and its impairment caused by AF conditions is the leading factor promoting hemodynamic conditions related to the thromboembolic risk. Hence, FSI models modeling active contraction and the interaction between tissues and pressurized blood are crucial to provide a better insight into the phenomenon.

Most importantly, no major differences were observed between the models in terms of critical flow parameters, indicating that the morphological class is not directly associated with the thromboembolic risk. Still, regions of very low SSR appeared to be concentrated in specific anatomical areas among the various models studied, in particular, in the lobes, after highly trabeculated regions, and in the areas characterized by sudden bends. These observations suggest that a different classification, based on local features rather than on the global gross shape of the LAA, may serve as a better descriptor of thromboembolic risks.

## Data Availability Statement

The raw data supporting the conclusions of this article will be made available by the authors, without undue reservation.

## Ethics Statement

This study was carried out in accordance with the recommendations of the South East Research Ethics Research Committee, Ayelsford, Kent, United Kingdom, with written informed consent from all subjects. All subjects gave written informed consent in accordance with the Declaration of Helsinki. The protocol was approved by the South East Research Ethics Research Committee, Ayelsford, Kent, United Kingdom. The patients/participants provided their written informed consent to participate in this study.

## Author Contributions

All authors was fully involved in the study and has contributed significantly to the submitted work, in terms of conception and design of the study, analysis and interpretation of the results, and critical review of the manuscript.

## Conflict of Interest

The authors declare that the research was conducted in the absence of any commercial or financial relationships that could be construed as a potential conflict of interest.

## Publisher’s Note

All claims expressed in this article are solely those of the authors and do not necessarily represent those of their affiliated organizations, or those of the publisher, the editors and the reviewers. Any product that may be evaluated in this article, or claim that may be made by its manufacturer, is not guaranteed or endorsed by the publisher.

## Supplementary Material

Supplementary Video 1 A cross-section video of the chicken wing model during a complete cardiac cycle, structural part.

Supplementary Video 2 A color-maps video of the maximum wall SSR during a complete cardiac cycle, fluid part.
